# Pulmonary Sarcoidosis Presenting as a Solitary Pulmonary Nodule With Probable Neurosarcoidosis: A Case Report

**DOI:** 10.1002/rcr2.70814

**Published:** 2026-11-03

**Authors:** Mai Homochi, Toshihiro Shirai, Toyohiro Sato, Aya Mukai, Tatsuya Tanaka, Fumitaka Hashiba, Ayako Masaki, Aya Naiki‐Ito, Osamu Takakuwa, Yoshihiro Kanemitsu

**Affiliations:** ^1^ Department of Education and Training Shizuoka General Hospital Shizuoka Japan; ^2^ Department of Respiratory Medicine Shizuoka General Hospital Shizuoka Japan; ^3^ Department of Neurology and Neuroscience Nagoya City University Graduate School of Medical Sciences Nagoya Aichi Japan; ^4^ Department of Respiratory Medicine, Allergy, and Clinical Immunology Nagoya City University Graduate School of Medical Sciences Nagoya Aichi Japan; ^5^ Department of Pathology and Molecular Diagnostics Nagoya City University Graduate School of Medical Sciences Nagoya Aichi Japan; ^6^ Department of Experimental Pathology and Tumor Biology Nagoya City University Graduate School of Medical Sciences Nagoya Aichi Japan; ^7^ Department of Medical Education Nagoya City University Graduate School of Medical Sciences Nagoya Aichi Japan

**Keywords:** lung cancer, neurosarcoidosis, paraneoplastic neurological syndrome, sarcoidosis, sensory neuronopathy

## Abstract

A 51‐year‐old woman presented with a solitary pulmonary nodule, hilar and mediastinal lymphadenopathy and progressive sensory disturbances. FDG‐PET demonstrated intense uptake in the pulmonary and hilar lesions, while spinal MRI showed thoracic cord enhancement. Lung cancer‐associated paraneoplastic neurological syndrome was initially suspected. Biopsies of the endobronchial lesion and a mediastinal lymph node revealed noncaseating granulomas without malignancy. Ocular involvement and neurological findings supported pulmonary sarcoidosis with probable neurosarcoidosis. Corticosteroid therapy improved neurological symptoms. Long‐term follow‐up showed a stable pulmonary nodule and sustained neurological improvement. This case highlights the importance of tissue diagnosis and careful neurological assessment when pulmonary nodules and sensory disturbances suggest malignancy.

## Graphical abstract

Pulmonary sarcoidosis presenting with a solitary pulmonary nodule and probable neurosarcoidosis can resemble lung cancer‐associated paraneoplastic neurological syndrome. Biopsies of a right B3b endobronchial lesion and a mediastinal lymph node revealed noncaseating granulomas, while neurological assessment supported probable neurosarcoidosis.

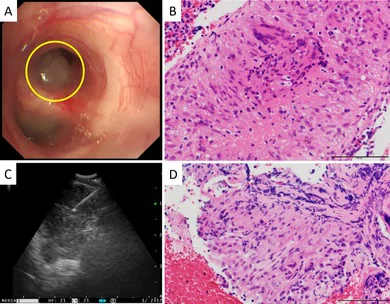

## 1 Introduction

Sarcoidosis is a multisystem granulomatous disease characterised by noncaseating granulomas, with pulmonary involvement in most patients. Although bilateral hilar lymphadenopathy is a common thoracic manifestation, nodular pulmonary sarcoidosis is uncommon and may closely mimic primary lung cancer on computed tomography (CT) and fluorodeoxyglucose positron emission tomography (FDG‐PET) [1]. Neurological involvement may further complicate diagnosis [2]. We report a patient with pulmonary sarcoidosis and an unbiopsied solitary pulmonary nodule, accompanied by probable neurosarcoidosis, initially raising suspicion for lung cancer‐associated paraneoplastic neurological syndrome.

## 2 Case Report

A 51‐year‐old never‐smoking woman with a family history of lung cancer was referred to Nagoya City University Hospital because of progressive neurological symptoms and chest imaging findings suspicious for malignancy. Her medical history included Sjögren syndrome, Hashimoto thyroiditis and depression. She developed fever, dysuria, fatigue and numbness over the lower back and left lateral thigh, followed by sensory disturbances involving the left chest and abdomen and paresthesia of both fingertips. Chest radiography demonstrated right hilar enlargement (Figure 1A), and CT revealed an 8‐mm solitary pulmonary nodule in the right upper lobe (S3), with right hilar and mediastinal lymphadenopathy (Figure 1B,C).

**FIGURE 1:**
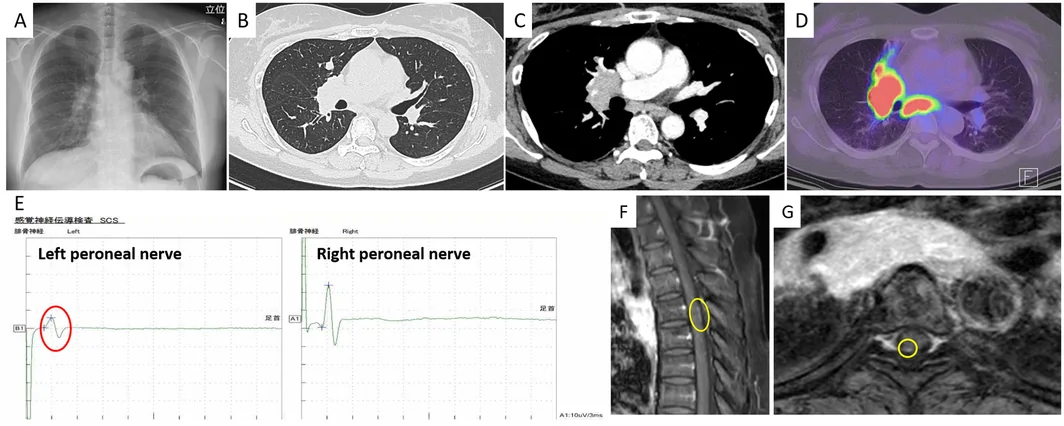
Clinical and imaging findings. (A) Chest radiography showing right hilar enlargement. (B, C) Chest CT showing an 8‐mm right S3 pulmonary nodule and right hilar and mediastinal lymphadenopathy. (D) FDG‐PET showing uptake in the S3 nodule and contiguous right hilar lesion, measured together (SUVmax, 42.2) and station 7 lymph node (SUVmax, 18.6). (E) Nerve conduction studies showing reduced sensory nerve action potential amplitude in the left peroneal nerve (red circle) compared with the right peroneal nerve. (F, G) Contrast‐enhanced spinal MRI showing linear gadolinium enhancement along the dorsal aspect of the thoracic spinal cord at Th6 (yellow circles); (F) sagittal and (G) axial images.

Neurological examination showed preserved muscle strength, normal deep tendon reflexes, and no cranial nerve abnormalities. Sensory examination revealed hypesthesia in the Th6–8 dermatomes, left scapular region and left lateral thigh, mild hypoalgesia in the Th11–12 dermatomes and subtle impairment of vibration sensation in both lower extremities. Serum soluble interleukin‐2 receptor was mildly elevated (692 U/mL), whereas angiotensin‐converting enzyme, calcium, lysozyme and lactate dehydrogenase were normal. Tumour markers, including carcinoembryonic antigen, CYFRA, squamous cell carcinoma antigen, pro‐gastrin‐releasing peptide and neuron‐specific enolase, were normal.

FDG‐PET demonstrated intense uptake in the S3 nodule and contiguous right hilar lesion, measured together (SUVmax, 42.2) and in the subcarinal lymph node (station 7; SUVmax, 18.6) (Figure 1D). Nerve conduction studies showed reduced sensory nerve action potential amplitude in the left peroneal nerve (Figure 1E). Contrast‐enhanced spinal magnetic resonance imaging revealed linear gadolinium enhancement along the dorsal aspect of the thoracic spinal cord at Th6 (Figure 1F,G); brain imaging was normal. Cerebrospinal fluid analysis showed mononuclear‐predominant pleocytosis (7 cells/μL), a protein level of 41 mg/dL, IgG index 0.54 and negative oligoclonal bands. Given the pulmonary lesion and subacute progressive sensory disturbances, paraneoplastic neurological syndrome, particularly sensory neuronopathy, was initially considered [3]. Sjögren‐associated neuropathy was another differential diagnosis.

Bronchoscopy revealed a nodular endobronchial lesion at right B3b and its transbronchial biopsy demonstrated noncaseating epithelioid granulomas without malignancy (Figure 2A,B). The S3 nodule was inaccessible by bronchoscopy. Acid‐fast staining was negative, and periodic acid–Schiff/Alcian blue staining identified no fungal organisms. An interferon‐gamma release assay was negative, and serum β‐d‐glucan was 4.0 pg/mL. Endobronchial ultrasound‐guided transbronchial needle aspiration of the station 7 lymph node also demonstrated noncaseating granulomas (Figure 2C,D). Ophthalmological examination identified iris nodules. Anti‐SS‐A/Ro and anti‐SS‐B/La antibodies were negative, without active glandular or extraglandular manifestations of Sjögren syndrome. Neuronal antibody testing was not performed because histopathological confirmation of systemic sarcoidosis had already been obtained. Spinal cord biopsy was avoided because of its invasiveness and potential neurological risks. Neurological findings and biopsy‐proven systemic sarcoidosis supported probable neurosarcoidosis according to consensus criteria [2].

**FIGURE 2:**
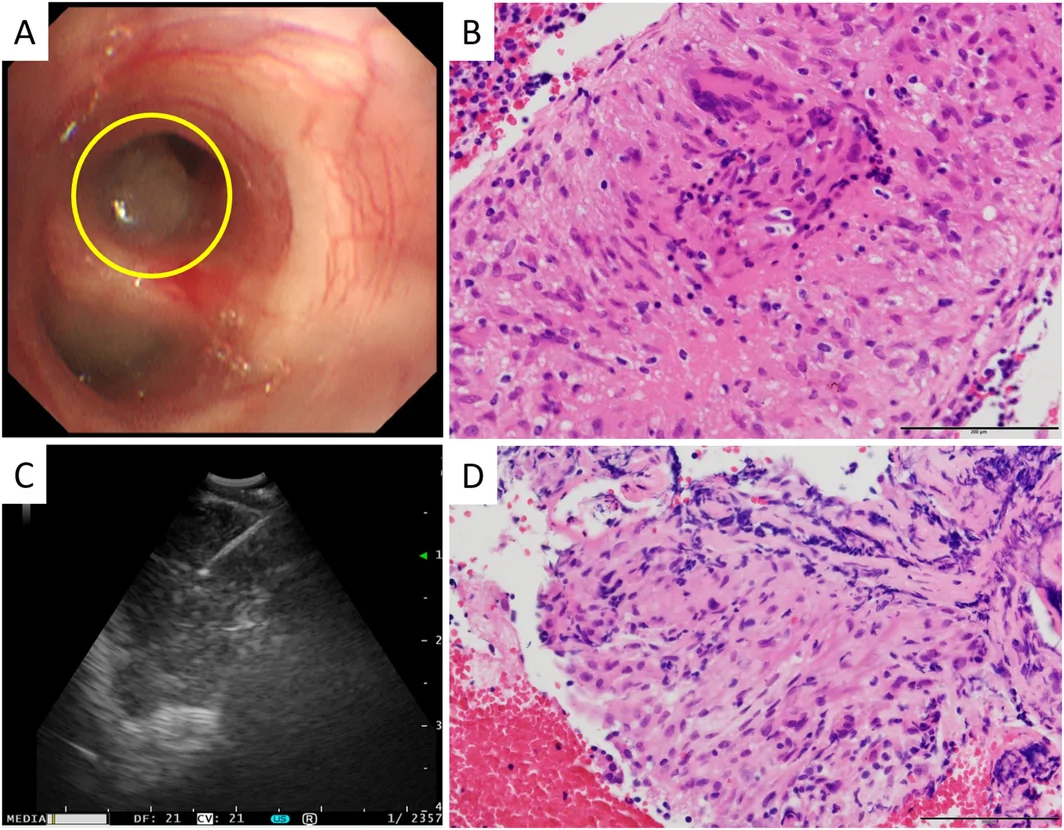
Bronchoscopic and histopathological findings. (A) Bronchoscopy showing a right B3b nodular lesion (yellow circle). (B) Transbronchial biopsy showing noncaseating epithelioid granulomas. (C) EBUS‐guided needle aspiration of the subcarinal lymph node (station 7). (D) Lymph node biopsy showing noncaseating granulomas. (B, D) Original magnification ×200, scale bar: 200 μm. Both specimens were evaluated by two pathology specialists.

Intravenous methylprednisolone (1000 mg/day for 3 days) was administered, followed by oral prednisolone, with gradual neurological improvement. At approximately 17 months after treatment initiation, chest CT showed a stable S3 nodule and decreased hilar lymphadenopathy. At approximately 21 months, gait and muscle strength were normal, upper‐extremity paresthesia had resolved and mild left lumbar tactile hypesthesia persisted.

## 3 Discussion

This case illustrates how a solitary pulmonary nodule in a patient with systemic sarcoidosis and neurological manifestations can mimic lung cancer‐associated paraneoplastic neurological syndrome. Nodular pulmonary sarcoidosis may resemble malignancy on CT and FDG‐PET [1]. In our patient, intense FDG uptake and progressive sensory disturbances raised suspicion for lung cancer. Histopathological examination of the endobronchial nodular lesion and mediastinal lymph node was therefore essential. Noncaseating granulomas, ocular involvement and evaluation for mycobacterial and fungal infection supported systemic sarcoidosis [4]. Normal serum angiotensin‐converting enzyme did not exclude this diagnosis [4].

The Neurosarcoidosis Consortium Consensus Group criteria require compatible neurological findings, evaluation of alternative causes and histopathological confirmation of systemic sarcoidosis for probable neurosarcoidosis [2]. Thoracic cord enhancement and sensory nerve abnormalities supported this classification, although neither were specific. Corticosteroid response was not diagnostic. These findings suggested possible central and peripheral nervous system involvement, although the underlying cause of the peripheral sensory abnormality remained uncertain.

Paraneoplastic sensory neuronopathy was initially suspected because of subacute sensory symptoms and the suspicious pulmonary lesion; it is a high‐risk phenotype under PNS‐Care criteria [3]. Sjögren syndrome may cause sensory ganglionopathy [5]. Negative anti‐SS‐A/Ro and anti‐SS‐B/La antibodies and absent active glandular symptoms could not exclude Sjögren‐associated neuropathy. However, thoracic spinal cord enhancement suggested central involvement not readily explained by isolated peripheral neuropathy, while biopsy‐proven systemic sarcoidosis favoured probable neurosarcoidosis. Coexisting neurological disease remained possible.

Mild cerebrospinal fluid pleocytosis was nonspecific. The absence of neuronal antibody testing limited the evaluation of alternative neurological causes, particularly paraneoplastic neurological syndrome. The lack of spinal cord biopsy also prevented direct neural tissue confirmation. The unbiopsied S3 nodule was clinically considered related to the biopsy‐proven B3b sarcoid lesion, but its histopathological nature remained uncertain. Its stability and decreased hilar lymphadenopathy during follow‐up were reassuring, although malignancy could not be excluded. Continued radiological surveillance remains appropriate.

In conclusion, sarcoidosis should be considered when pulmonary nodules and progressive neurological symptoms suggest lung cancer‐associated paraneoplastic neurological syndrome. Histopathological evaluation and careful neurological assessment are crucial for distinguishing these conditions.

## Author Contributions

**Mai Homochi:** writing – original draft, writing – review and editing, visualization, investigation, data curation. **Toshihiro Shirai:** project administration, supervision, writing – review and editing. **Toyohiro Sato:** investigation. **Aya Mukai:** investigation. **Tatsuya Tanaka:** investigation. **Fumitaka Hashiba:** investigation. **Ayako Masaki:** investigation, resources. **Aya Naiki‐Ito:** investigation, resources. **Osamu Takakuwa:** supervision. **Yoshihiro Kanemitsu:** data curation, resources, writing – review and editing, supervision.

## Funding

The authors have nothing to report.

## Ethics Statement

Ethics approval was not required for this single‐patient case report according to institutional policy.

## Consent

The authors declare that written informed consent was obtained for the publication of this manuscript and accompanying images using the consent form provided by the Journal.

## Conflicts of Interest

The authors declare no conflicts of interest.

## Data Availability

The data that support the findings of this study are available on request from the corresponding author. The data are not publicly available due to privacy or ethical restrictions.
